# Supplementary material for: Cancer Treatment Patterns and Factors Affecting Receipt of Treatment in Older Adults: Results from the ASPREE Cancer Treatment Substudy (ACTS)
Source: Cancers (Basel). 2023 Feb 5;15(4):1017. doi: 10.3390/cancers15041017 (PMC9953887; doi:10.3390/cancers15041017)
Supplement: Supplementary file 1 [file cancers-15-01017-s001.zip › cancers-2109987-supplementary.pdf]

Supplementary Figure S1. Flow diagram of inclusion for the ASPREE Cancer Treatment Substudy (ACTS).

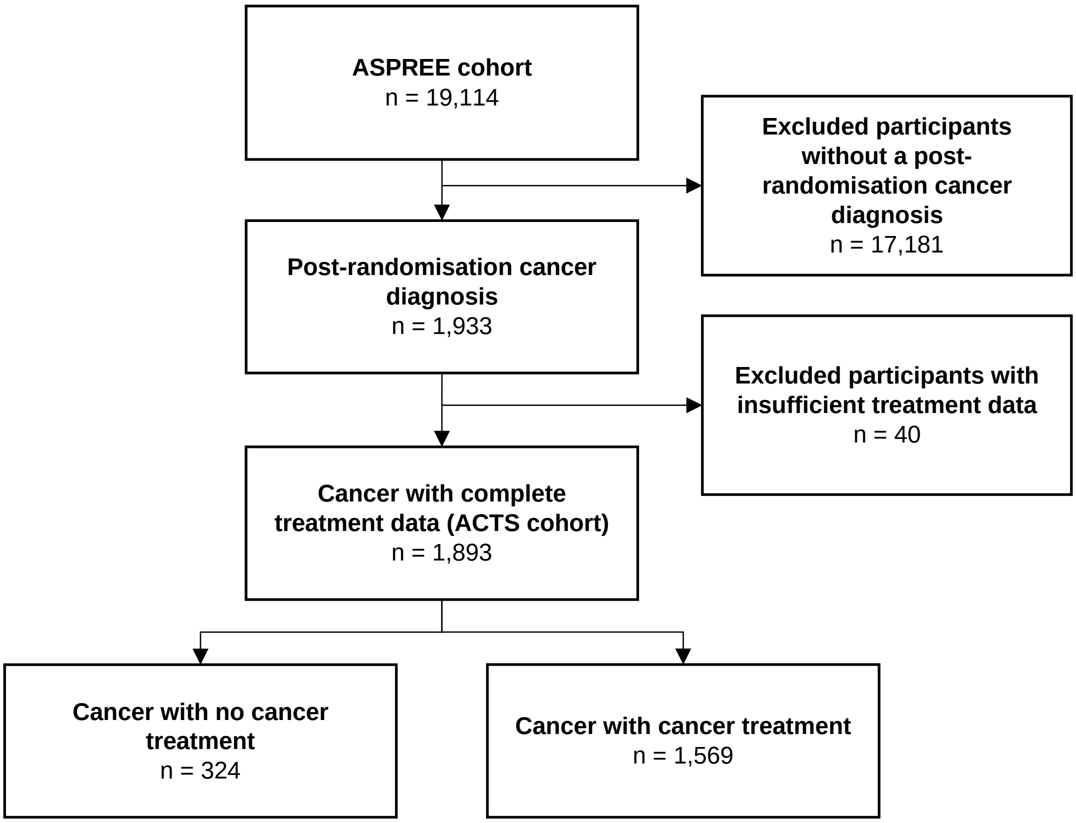

Supplementary Table S1. Coding criteria for cancer treatment data, stratified by treatment modality.

**Type of cancer treatment data**	**Criteria**
Systemic therapy<br>- Cytotoxic chemotherapy<br>- Hormonal therapy<br>- Targeted therapy<br>- Immunotherapy	*Inclusion*<br>• Any systemic therapy including, but not limited to, cytotoxic chemotherapy, hormonal therapy, targeted therapy, and immunotherapy<br>*Exclusion*<br>• Regional chemotherapy *e.g.* transarterial chemoembolisation, hypothermic intraperitoneal chemotherapy, intravesical mitomycin c<br>• Regional immunotherapy *e.g.* intravesical BCG (Bacillus Calmette-Guerin) immunotherapy (early-stage bladder cancer)<br>*Miscellaneous*<br>• Arsenic trioxide + all-trans retinoic acid for acute promyelocytic leukaemia should be coded as targeted therapy
Radiation therapy	*Inclusion*<br>• Any radiation therapy procedure used in the treatment of a primary tumour or its metastases<br>*Exclusions*<br>• None<br>*Miscellaneous*<br>• Radioactive iodine for thyroid cancer should be coded as radiation therapy
Surgery	*Inclusion*<br>• Any operation that has been undertaken to remove a tumour *e.g.* anterior resection for colorectal cancer, mastectomy for breast cancer, wide local excisions of melanoma, endoscopic resection of a known tumour<br>• Any operation that has been undertaken to debulk the tumour<br>• Any operation related to the treatment of the tumour or its associated symptoms *e.g.* video-assisted thoracoscopic pleurodesis for pleural effusion secondary to lung cancer, transurethral resection of the prostate for prostatomegaly secondary to prostate cancer, bypass surgery for pancreatic surgery<br>*Exclusion*<br>• Endoscopic procedures without intent for curative resection of a tumour *e.g.* colonoscopy, cystoscopy, bronchoscopy<br>• Biopsies and other diagnostic procedures *e.g.* excision biopsies for melanoma or breast cancer<br>• Procedures in which a cancer was incidentally discovered *e.g.* diagnosis of prostate cancer on transurethral resection of the prostate for benign prostatic hyperplasia
Regional therapy	*Inclusion*

	• Regional chemotherapy *e.g.* transarterial chemoembolisation, hypothermic intraperitoneal chemotherapy, intravesical mitomycin c<br>• Regional immunotherapy for confirmed cancers *e.g*. intravesical BCG (Bacillus Calmette-Guerin) immunotherapy<br>*Exclusion*<br>• Regional therapy for in-situ cancers *e.g.* intravesical BCG for bladder carcinomas in-situ

Supplementary Table S2. Cancer treatment modalities received for other incident post-randomisation cancers during ASPREE, stratified by cancer type and metastatic status, and cause of death and time from diagnosis to death.

		Systemic Therapy						
	**Any treatment (n=1,569)**	**Any systemic therapy (n=869)**	**Chemotherapy (n=537)**	**Hormonal therapy (n=351)**	**Targeted therapy (n=85)**	**Immunotherapy (n=31)**	**Radiation therapy (n=544)**	**Surgery (n=1,029)**
**Non-metastatic solid tumours**								
Bladder (n=59)	48 (81%)	15 (25%)	15 (25%)	-	-	1 (2%)	9 (15%)	32 (54%)
Brain (n=25)	17 (68%)	8 (32%)	8 (32%)	-	-	-	10 (40%)	16 (64%)
Cervical (n=3)	3 (100%)	3 (100%)	3 (100%)	-	-	-	3 (100%)	-
Gallbladder or bile duct (n=11)	6 (55%)	4 (36%)	4 (36%)	-	-	-	2 (18%)	3 (27%)
Kidney (n=25)	24 (96%)	1 (4%)	-	-	1 (4%)	-	1 (4%)	24 (96%)
Liver (n=3)	2 (67%)	2 (67%)	-	-	2 (67%)	1 (33%)	1 (33%)	1 (33%)
Mesothelioma (n=14)	12 (86%)	6 (43%)	6 (43%)	-	-	-	3 (21%)	8 (57%)
Oesophageal (n=14)	13 (93%)	8 (57%)	8 (57%)	-	-	-	11 (79%)	5 (36%)
Ovary or endometrium (n=39)	36 (92%)	12 (31%)	11 (28%)	1 (3%)	-	-	15 (38%)	35 (90%)
Pancreas (n=31)	23 (74%)	14 (45%)	14 (45%)	-	-	-	2 (6%)	18 (58%)
Stomach (n=21)	17 (81%)	8 (38%)	8 (38%)	-	-	-	5 (24%)	16 (76%)
Thyroid (n=10)	10 (100%)	-	-	-	-	-	6 (60%)	9 (90%)
**Metastatic solid tumours**								
Bladder (n=10)	7 (70%)	4 (40%)	4 (40%)	-	-	-	3 (30%)	2 (20%)
Brain (n=0)	N/A	N/A	N/A	N/A	N/A	N/A	N/A	N/A
Cervical (n=0)	N/A	N/A	N/A	N/A	N/A	N/A	N/A	N/A
Gallbladder or bile duct (n=10)	5 (50%)	5 (50%)	5 (50%)	-	-	-	-	1 (10%)
Kidney (n=10)	7 (70%)	3 (30%)	2 (20%)	-	1 (10%)	-	4 (40%)	4 (40%)
Liver (n=4)	3 (75%)	1 (25%)	-	-	1 (25%)	-	1 (25%)	1 (25%)
Mesothelioma (n=1)	1 (100%)	-	-	-	-	-	-	1 (100%)

Oesophageal (n=13)	11 (85%)	9 (69%)	9 (69%)	-	-	-	9 (69%)	-
Ovary or endometrium (n=34)	28 (82%)	24 (71%)	23 (68%)	2 (6%)	3 (9%)	-	2 (6%)	18 (53%)
Pancreas (n=33)	20 (61%)	16 (48%)	16 (48%)	-	-	-	2 (6%)	7 (21%)
Stomach (n=8)	6 (75%)	2 (25%)	2 (25%)	-	-	-	3 (38%)	1 (13%)
Thyroid (n=0)	N/A	N/A	N/A	N/A	N/A	N/A	N/A	N/A
Percentages represent the proportion of participants who received cancer treatment relative to the first column. For example, 81% of participants with bladder cancers received any cancer treatment.<br>“-“ indicates that no one (0 participants) in this group received a particular treatment modality.								

Supplementary Table S3. Cancer treatment modalities received for common non-sex specific incident post-randomisation cancers during ASPREE, stratified by cancer type and sex (F = female, M = male).

			**Systemic Therapy**											
	**Any treatment (n=1,569)**		**Any systemic therapy (n=869)**		**Chemotherapy (n=537)**		**Targeted therapy (n=85)**		**Immunotherapy (n=31)**		**Radiation therapy (n=544)**		**Surgery (n=1,029)**	
	F	*M*	*F*	*M*	*F*	*M*	*F*	*M*	*F*	*M*	*F*	*M*	*F*	*M*
**Non-metastatic solid tumours**														
Colorectal (n_F=98; n_m=108)	90 (92%)	101 (94%)	30 (31%)	41 (38%)	30 (31%)	41 (38%)	1 (1%)	3 (3%)	-	-	7 (7%)	13 (12%)	98 (100%)	98 (91%)
Lung (n_F=31; n_m=44)	29 (94%)	36 (82%)	10 (32%)	10 (23%)	9 (29%)	9 (20%)	-	1 (2%)	2 (6%)	-	14 (45%)	19 (43%)	16 (52%)	22 (50%)
Melanoma (n_F=60; n_m=100)	57 (95%)	94 (94%)	3 (5%)	2 (2%)	-	-	-	-	3 (5%)	2 (2%)	1 (2%)	4 (4%)	55 (92%)	91 (91%)
**Metastatic solid tumours**														
Colorectal (n_F=26; n_m=30)	23 (88%)	27 (90%)	20 (77%)	22 (73%)	20 (77%)	22 (73%)	12 (46%)	8 (27%)	-	-	4 (15%)	7 (23%)	14 (54%)	21 (70%)
Lung (n_F=26; n_m=51)	20 (77%)	39 (76%)	12 (46%)	27 (53%)	9 (35%)	27 (53%)	3 (12%)	3 (6%)	2 (8%)	4 (8%)	16 (62%)	24 (47%)	5 (19%)	10 (20%)
Melanoma (n_F=10; n_m=18)	8 (80%)	15 (83%)	3 (30%)	12 (67%)	1 (10%)	1 (6%)	1 (10%)	4 (22%)	1 (10%)	10 (56%)	3 (30%)	7 (39%)	5 (50%)	11 (61%)
**Haematological malignancy** (n_F=97; n_m=88)	64 (66%)	48 (55%)	58 (60%)	44 (50%)	54 (56%)	43 (49%)	10 (10%)	9 (10%)	-	2 (2%)	10 (10%)	6 (7%)	6 (6%)	4 (5%)

Percentages represent the proportion of participants who received cancer treatment relative to the first column. For example, 92% of female participants with non-metastatic colorectal cancers received any cancer treatment.
"-" indicates that no one (0 participants) in this group received a particular treatment modality.

Supplementary Table S4. Cancer treatment modalities received for common incident post-randomisation cancers during ASPREE, stratified by cancer type and country of residence (Aus = Australia, US = United States).

			**Systemic Therapy**													
	**Any treatment (n=1,569)**		**Any systemic therapy (n=869)**		**Chemotherapy (n=537)**		**Hormonal therapy (n=351)**		**Targeted therapy (n=85)**		**Immunotherapy (n=31)**		**Radiation therapy (n=544)**		**Surgery (n=1,029)**	
	Aus	*US*	*Aus*	*US*	*Aus*	*US*	*Aus*	*US*	*Aus*	*US*	*Aus*	*US*	*Aus*	*US*	*Aus*	*US*
**All cancers** **(**n_{Aus}=1,758; n_{US}=175)	1,444 (82%)	125 (71%)	799 (45%)	70 (40%)	501 (28%)	36 (21%)	317 (18%)	34 (19%)	82 (5%)	3 (2%)	27 (2%)	4 (2%)	512 (29%)	32 (18%)	944 (54%)	85 (49%)
**Non-metastatic solid tumours**																
Breast (n_{Aus}=181; n_{US}=30)	181 (100%)	29 (97%)	150 (83%)	21 (70%)	46 (25%)	3 (10%)	129 (71%)	20 (67%)	13 (7%)	-	-	-	97 (54%)	7 (23%)	179 (99%)	27 (90%)
Colon/rectum (n_{Aus}=198; n_{US}=8)	185 (93%)	6 (75%)	68 (34%)	3 (38%)	68 (34%)	3 (38%)	N/A	N/A	4 (2%)	-	-	-	19 (10%)	1 (13%)	182 (92%)	5 (63%)
Lung (n_{Aus}=68; n_{US}=7)	60 (88%)	5 (71%)	20 (29%)	-	18 (26%)	-	N/A	N/A	1 (1%)	-	2 (3%)	-	33 (49%)	-	33 (49%)	5 (71%)
Melanoma (n_{Aus}=154; n_{US}=6)	149 (97%)	2 (33%)	4 (3%)	1 (17%)	-	-	N/A	N/A	-	-	4 (3%)	1 (17%)	5 (3%)	-	145 (94%)	1 (17%)
Prostate (n_{Aus}=248; n_{US}=18)	182 (73%)	9 (50%)	94 (38%)	4 (22%)	5 (2%)	-	92 (37%)	4 (22%)	-	-	-	-	102 (41%)	4 (22%)	79 (32%)	4 (22%)
**Metastatic solid tumours**																
Breast (n_{Aus}=27; n_{US}=5)	24 (89%)	4 (80%)	22 (81%)	4 (80%)	8 (30%)	2 (40%)	17 (63%)	3 (60%)	4 (15%)	-	-	-	12 (44%)	3 (60%)	7 (26%)	1 (20%)
Colon/rectum (n_{Aus}=54; n_{US}=2)	49 (91%)	1 (50%)	41 (76%)	1 (50%)	41 (76%)	1 (50%)	N/A	N/A	20 (37%)	-	-	-	11 (20%)	-	35 (65%)	-
Lung (n_{Aus}=68; n_{US}=9)	54 (79%)	5 (56%)	36 (53%)	3 (33%)	33 (49%)	3 (33%)	N/A	N/A	6 (9%)	-	4 (6%)	2 (22%)	36 (53%)	4 (44%)	15 (22%)	-
Melanoma (n_{Aus}=25; n_{US}=3)	21 (84%)	2 (67%)	14 (56%)	1 (33%)	1 (4%)	1 (33%)	N/A	N/A	5 (20%)	-	11 (44%)	-	9 (36%)	1 (33%)	15 (60%)	1 (33%)
Prostate (n_{Aus}=87; n_{US}=11)	80 (92%)	8 (73%)	78 (90%)	8 (73%)	24 (28%)	1 (9%)	76 (87%)	7 (64%)	-	-	-	-	31 (36%)	4 (36%)	16 (18%)	2 (18%)
**Haematological malignancy** (n_{Aus}=164; n_{US}=21)	103 (63%)	9 (43%)	94 (57%)	8 (38%)	90 (55%)	7 (33%)	N/A	N/A	18 (11%)	1 (5%)	2 (1%)	-	14 (9%)	2 (10%)	10 (6%)	-

NB: Percentages represent the proportion of participants who received cancer treatment relative to the first column. For example, 100% of Australian participants with non-metastatic breast cancers received any cancer treatment.
"-" indicates that no one (0 participants) in this group received a particular treatment modality.
